# The 2026 *Salmonella Enteritidis* outbreak in the United Kingdom: Implications for food safety, imported-food surveillance, and supply-chain traceability

**DOI:** 10.1016/j.nmni.2026.101844

**Published:** 2026-09-09

**Authors:** Nasra Salad Ahmed, Fatima Abdijabbar Ibrahim, Omar Aweis Ali

**Affiliations:** a Faculty of Health Sciences and Tropical Medicine, Somali National University, Mogadishu, Somalia; b Climate and Environmental Research Institute (CERI), Mogadishu, Somalia; c Center for Natural Resources, PGRN, Universidade Estadual do Mato Grosso do Sul (UEMS), Dourados, Brazil

The ongoing *Salmonella Enteritidis* outbreak in the United Kingdom highlights the challenges of tracing foodborne infections across increasingly complex supply chains. As of August 13, 2026, the UK Health Security Agency (UKHSA) identified 207 confirmed cases-199 in England, six in Scotland, and one each in Wales and Northern Ireland all belonging to the same five-single-nucleotide-polymorphism cluster by whole-genome sequencing (WGS) [1]. Among cases with available information, 38% required hospital admission because of salmonellosis symptoms, two developed bloodstream infections, and one death was associated with the outbreak [1]. Epidemiological investigation found that 95 of 114 interviewed cases had eaten food prepared outside the home, while egg consumption remained the strongest food signal identified [1].

The possibility of egg-associated transmission is epidemiologically significant. European outbreak data have attributed approximately 33% of salmonellosis outbreaks with identified food sources to eggs, with *S. Enteritidis* outbreaks being particularly associated with this vehicle [2]. However, the current UK investigation requires careful interpretation: food-chain investigations have identified multiple egg suppliers, including importers, and the Food Standards Agency has reported potential links to eggs imported from outside the UK; however, a definitive source has not yet been established [1]. This distinction is important because epidemiological associations alone cannot establish that imported eggs caused the outbreak.

WGS has substantially strengthened the ability to distinguish *S. Enteritidis* outbreak isolates and identify genetically related cases that may otherwise appear epidemiologically disconnected [3]. Nevertheless, genomic relatedness does not independently identify the contaminated food; effective source attribution requires integration with exposure histories, microbiological evidence, and food-chain traceback [3,4]. Experience from multinational *Salmonella* outbreaks demonstrates that rapid sharing of epidemiological and genomic information between countries can accelerate the identification of internationally distributed food vehicles [4,5].

Three priorities emerge. First, surveillance of imported eggs and egg products should be risk-based and responsive to emerging epidemiological and food-chain signals [1,2]. Second, traceability systems should permit investigators to reconstruct product movement rapidly across food businesses, distributors, importers, and producers, particularly when supply chains cross national borders [4]. Third, human genomic surveillance should be integrated with epidemiological, food-safety, veterinary, and non-human microbiological evidence, accompanied by timely international data sharing [5].

The UK outbreak illustrates that early genomic detection, although essential, is only the beginning of effective outbreak control. Translating genomic signals into actionable source hypotheses requires rapid traceability, integrated surveillance, and coordinated cross-border investigation [4,5].

## CRediT authorship contribution statement

**Nasra Salad Ahmed:** Writing – original draft. **Fatima Abdijabbar Ibrahim:** Conceptualization. **Omar Aweis Ali:** Supervision.

## Declaration of competing interest

The authors declare that they have no known competing financial interests or personal relationships that could have appeared to influence the work reported in this paper.
